# Phase-field approach to evolution and interaction of twins in single crystal magnesium

**DOI:** 10.1007/s00466-022-02209-3

**Published:** 2022-07-27

**Authors:** Benhour Amirian, Hossein Jafarzadeh, Bilen Emek Abali, Alessandro Reali, James David Hogan

**Affiliations:** 1 Department of Mechanical Engineering, University of Alberta, Edmonton, T6G 2R3 AB Canada; 2 Department of Civil Engineering and Architecture, University of Pavia, I-27100 Pavia, Italy; 3 Department of Materials Science and Engineering, Uppsala University, 751 21 Uppsala, Sweden

**Keywords:** Phase-field model, Single crystal magnesium, Twinning interactions, Monolithic scheme

## Abstract

Crack initiation and propagation as well as abrupt occurrence of twinning are challenging fracture problems where the transient phase-field approach is proven to be useful. Early-stage twinning growth and interactions are in focus herein for a magnesium single crystal at the nanometer length-scale. We demonstrate a basic methodology in order to determine the mobility parameter that steers the kinetics of phase-field propagation. The concept is to use already existing molecular dynamics simulations and analytical solutions in order to set the mobility parameter correctly. In this way, we exercise the model for gaining new insights into growth of twin morphologies, temporally-evolving spatial distribution of the shear stress field in the vicinity of the nanotwin, multi-twin, and twin-defect interactions. Overall, this research addresses gaps in our fundamental understanding of twin growth, while providing motivation for future discoveries in twin evolution and their effect on next-generation material performance and design.

## Introduction

Developing next-generation materials with controlled twinning behaviors offers promising opportunities for improved mechanical properties [1, 2] and performance in engineering applications (e.g., gas turbine engines [3] and lightweight automotive structures [4]). Among materials that exhibit twinning [5–8], magnesium [9–12] is an example of a light-weight metal where slip and twinning, as the two main crystallographic mechanisms, play a decisive role in its mechanical response; here, twinning is favorable on pyramidal {1012} $\langle 1011\rangle$ systems at room temperature [13]. In magnesium, single twinning occurs through contraction [14] and extension strains [15] along the *c*-axis [16]. Recent twinning studies have focused on observations of asymmetric twin growth due to heterogeneous grain deformation in the vicinity of the twin [17, 18]. We understand that interaction of twin boundaries with other defects (i.e., voids and self-interstitials) increases the likelihood for void nucleation, cracking, and premature failure, leading to degradation of material performance and reduction of material lifetime [19, 20]. Recent efforts have also been made to model the twin local stress accurately by means of neighboring grains to accommodate the transformation [21]. In engineering applications, there is a broad interest in incorporating magnesium in high strain-rate applications (e.g., aerospace [22]), where twin growth and evolution limits the mechanical performance [23]. However, knowledge gaps in understanding twin growth [24], thickening [25], and interactions [26] need to be addressed before the adoption of magnesium-based alloys into these applications; these are studied herein for a single crystal Mg material system.

Ample experimental measurements exist on time-resolved twin evolution in magnesium [27]. In situ data is limited effected by the limitations in available diagnostics to capture growth and evolution behaviors at sufficient length and time scales [28]. To this end, atomistic simulations have been widely adopted to probe effects such as atomic shuffling mechanisms for propagation of twins in magnesium [29], disconnections and other defects associated with the twin interface [30], and reaction of lattice dislocations with twin boundaries [31]. While new understandings have been gained to accurately model plastic deformation and fracture in magnesium [32, 33], atomistic simulations are limited in their ability to simulate twinning behaviors at relevant length and time scales needed for practical implementation in engineering applications. Challenges also exist in molecular dynamic approaches in applying characterization algorithms (e.g., centrosymmetry parameter [34] and bond angle analysis [35]) to interpret post-deformation crystal structure defect types (e.g., twinning) [36]. Continuum mechanics modeling utilizing crystal plasticity theory is yet another modeling approach for predicting the twinning and de-twinning response in materials with hexagonal close-packed crystal structures [37–39]. However, crystal plasticity modeling has difficulties to capture the twinning process correctly due to treating the twinning deformation as a unidirectional shear deformation mode [40]. Additionally, the conventional crystal plasticity model is unable to investigate the effect of twin microstructure on the mechanical behavior of magnesium at the nanometer scale [41]. Overcoming such limitations, we model herein the twinning process by a phase-field approach where the mobility parameter is determined by an inverse analysis. Such a computational implementation allows us to unravel time-evolved twinning behavior in magnesium.

For the morphological evolution of twins, the mesoscale phase-field model [42–47] has been extensively used to study the nucleation [48], growth [49], and propagation of twinning [50]. Most recent computational approaches to phase field equations for studying deformation twinning in magnesium at the microscale were based on the Fourier spectral method [50–52]. However, such an approach is only applicable to cases involving periodic boundary conditions and for morphologies and microstructures dominated by long-range elastic interactions [53]. Also, spectral method is mostly used for solving linear problems [54]. In [52, 55, 56], the proposed phase-field simulations for deformation twinning and dislocation induced plasticity in hexagonal closed-pack materials were formulated on small strain theory; still, the twin evolution is usually accompanied by large interface orientation and large shear deformations [57] even under small strains [58]. Thus, coupling between twin evolution and fracture is of importance to achieve high accuracy in the numerical solution. In terms of validating the phase-field results of transmission mechanisms of deformation twins, atomistic simulations (e.g., molecular dynamics simulations [50, 55] and density functional theory [52]) and experimental results [51, 59] are the most widely used. Some drawbacks to these validations exist such as

- discrepancies of the peak stress value from the simulation and experimental data [51],
- qualitative comparison of distribution of order parameter using the isotropic gradient energy parameter [52, 55, 56],
- adopting empirically determined large non-physical values for the phase-field parameters (e.g., twin-twin interfacial energy, initial twin nucleus, and energy barrier heights between the matrix and the twinning [50, 51]), and
- validating at the different length-scales [50, 60].

Hence, the application of their model is somehow limited for studying the deformation mechanisms of Mg. The development of nanoscale phase-field models is therefore required and all the mentioned shortcomings are addressed in this work.

Building on these past works, this current article utilizes a monolithically-solved finite element method for solving an advanced physics-based phase-field approach to study the nanoscale growth of existing twins in anisotropic single crystal magnesium. We follow [61] for modeling the twinning interface propagation kinetics, which is important for the realistic description of twinning deformation. The model sheds light on the growth and evolving of twinning embryo.

A finite size sample with a hole is considered for studying the interactions of twin with defects, without the need of periodic boundaries. We implement nonlinear elasticity coupled to Ginzburg–Landau equations for order parameters. By using a highly nonlinear phase-field approach, we model anisotropic surface energy allowing to simulate large deformation of defect-free volumes at the nanoscale. Motivated by the literature [62–66], we use a mobility parameter and devote the work for determining this value for a specific material, namely single crystal Mg. The time evolution of the twin order parameter is directly proportional to the resolved shear stress. This outcome is useful for modeling deformation twinning since the propagation speed of twin boundaries is difficult to measure experimentally, and could even be supersonic if the driving stress is sufficiently large [67].

**Fig. 1:**
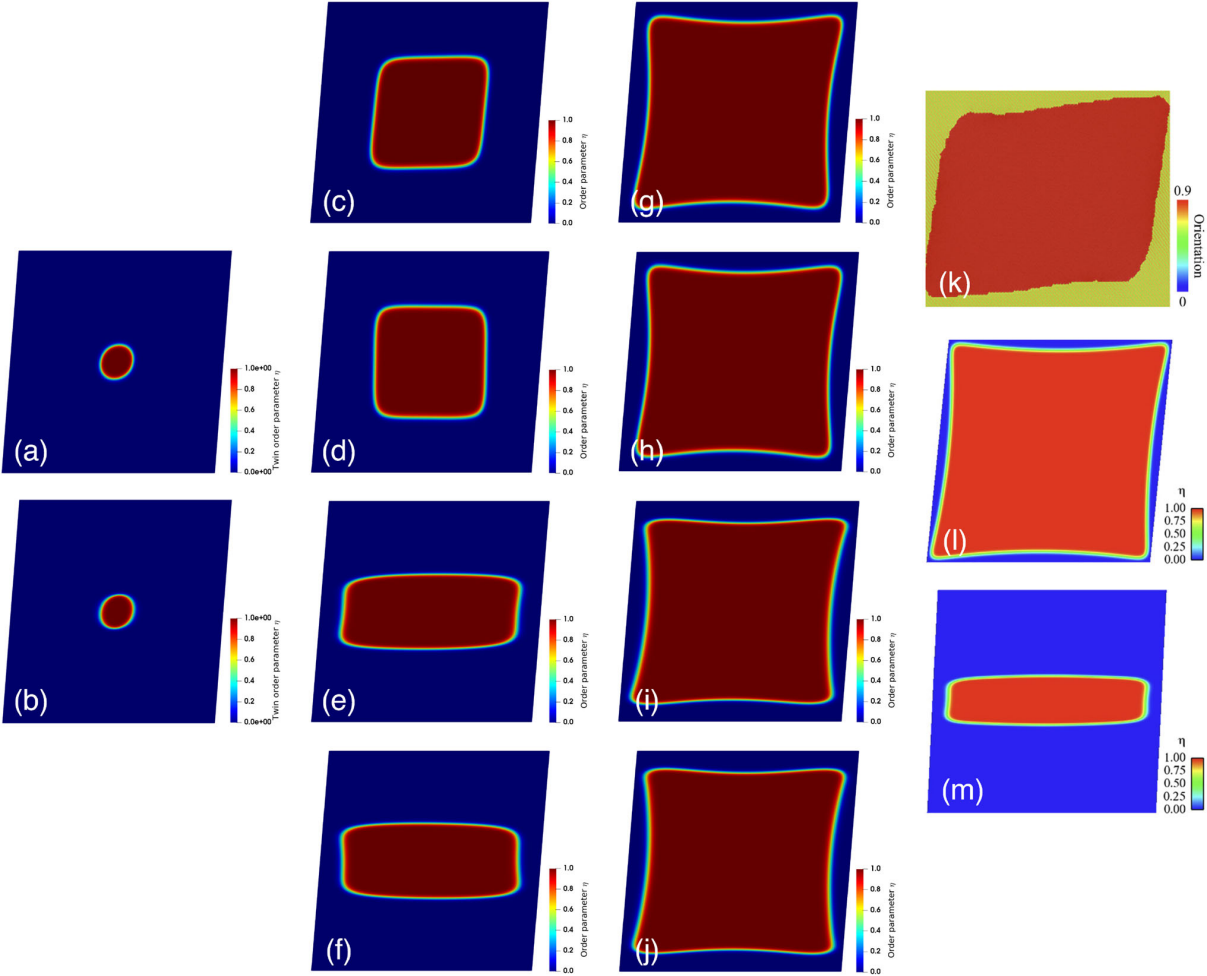
Distribution of the twin order parameter, $\eta$, for an initially circular single twin with radius of $3 \text { nm}$ in a simple-sheared rectangular domain in both small and large deformations considering both isotropic and anisotropic surface energy and elasticity with zero orientation of the habit plane. The initial conditions are chosen to match results published in the literature using a static phase-field approach [68] and molecular dynamics model [69], while the choice of times are selected to show the evolution of the twin growth under noted conditions. (a,b) Twin order parameter for small and large strains with an isotropic surface energy at $t=1 \text { ps}$; (c,d) Twin order parameter for small and large strains and isotropic surface energy at $t=50 \text { ps}$; (e,f) Twin order parameter for small and large strains and anisotropic surface energy at $t=50 \text { ps}$; (g,h) Twin order parameter for small and large strains and isotropic surface energy at $t=500 \text { ps}$; (i,j) Twin order parameter for small and large strains and anisotropic surface energy at $t=500 \text { ps}$; (k) Local orientation of the twinned region obtained from molecular dynamics simulations [69] and used to contrast with (g) and (h); (l,m) Order parameter for both isotropic and anisotropic surface energy under simple shear loading using a phase-field model from the literature [68], to be compared with (e) and (g). (k) and (l,m) are reproduced with permission from [68] and [69], respectively. (For interpretation of the references to color in this figure, the reader is referred to the web version of this article.)

**Fig. 2:**
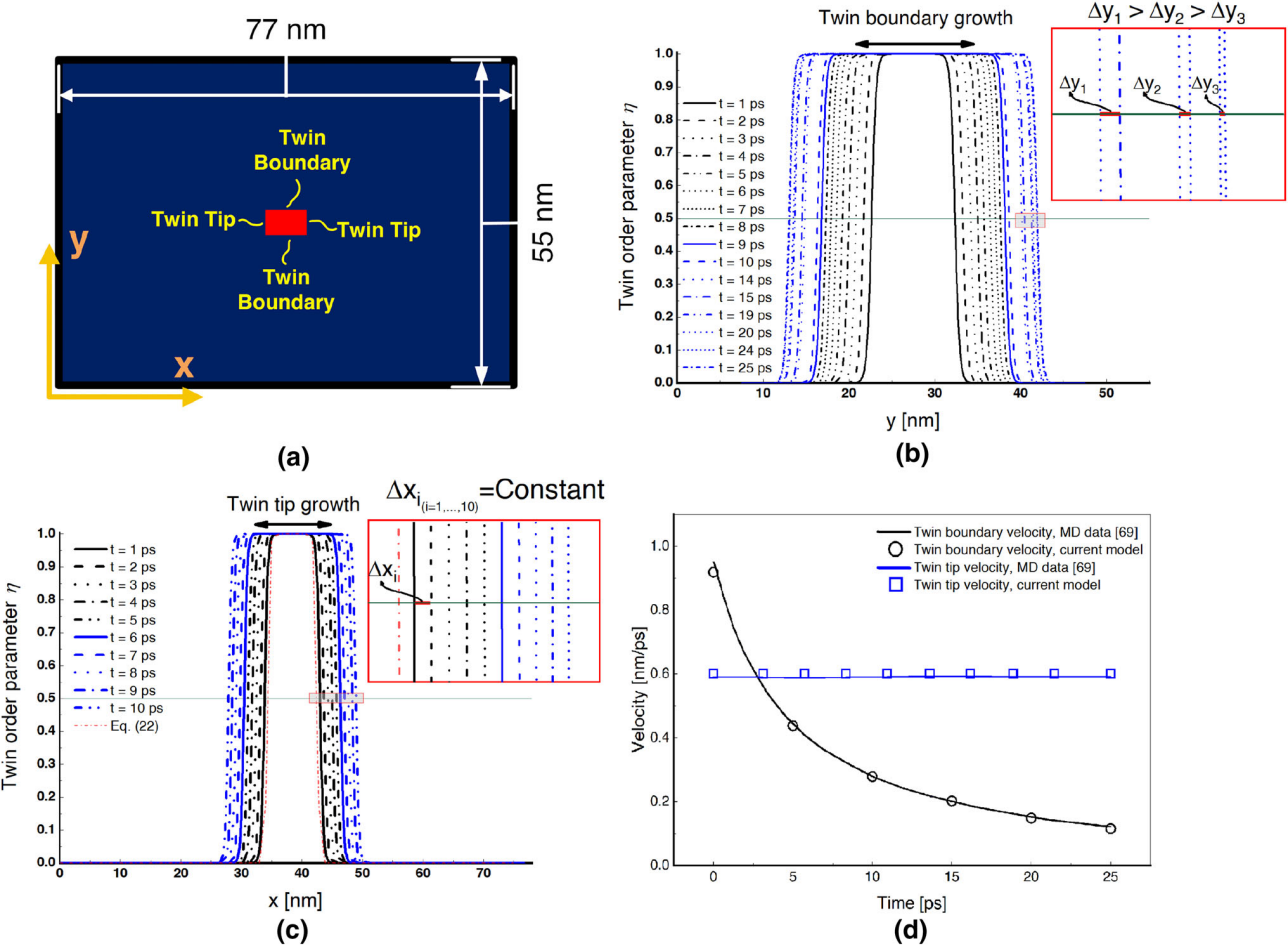
Evolution of twin growth in single-crystal pure magnesium. (a) Numerical setup of the rectangular single crystal with an initial rectangular twin with boundaries and tips in material configuration; (b) Time evolution of the twin order parameter as a function of the position *y* normal to the habit plane. A horizontal line starting from point $\eta = 0.5$ is chosen for measuring the twin boundary interface velocity to show the interface displacement $\Delta y$. The inset demonstrates the interface profile at six different time instants to show the time-dependent growth of the twin; (c) Time evolution of the twin order parameter as a function of the position *x* in the direction of the habit plane. Fewer time instants than shown in (b) are used to demonstrate the constant twin tip interface velocity. Similarly, the point $\eta = 0.5$ is chosen for measuring the tip interface velocity and to show the constant interface displacement $\Delta x$. The analytical solution of the explicit Ginzburg–Landau equation, which corresponds to $t = 0 \text { ps}$, is shown as the dotted red color; (d) Twin tip and twin boundary velocities as a function of time obtained from (b) and (c), and compared with those from molecular dynamics simulations [69]. (For interpretation of the references to color in this figure, the reader is referred to the web version of this article.)

**Fig. 3:**
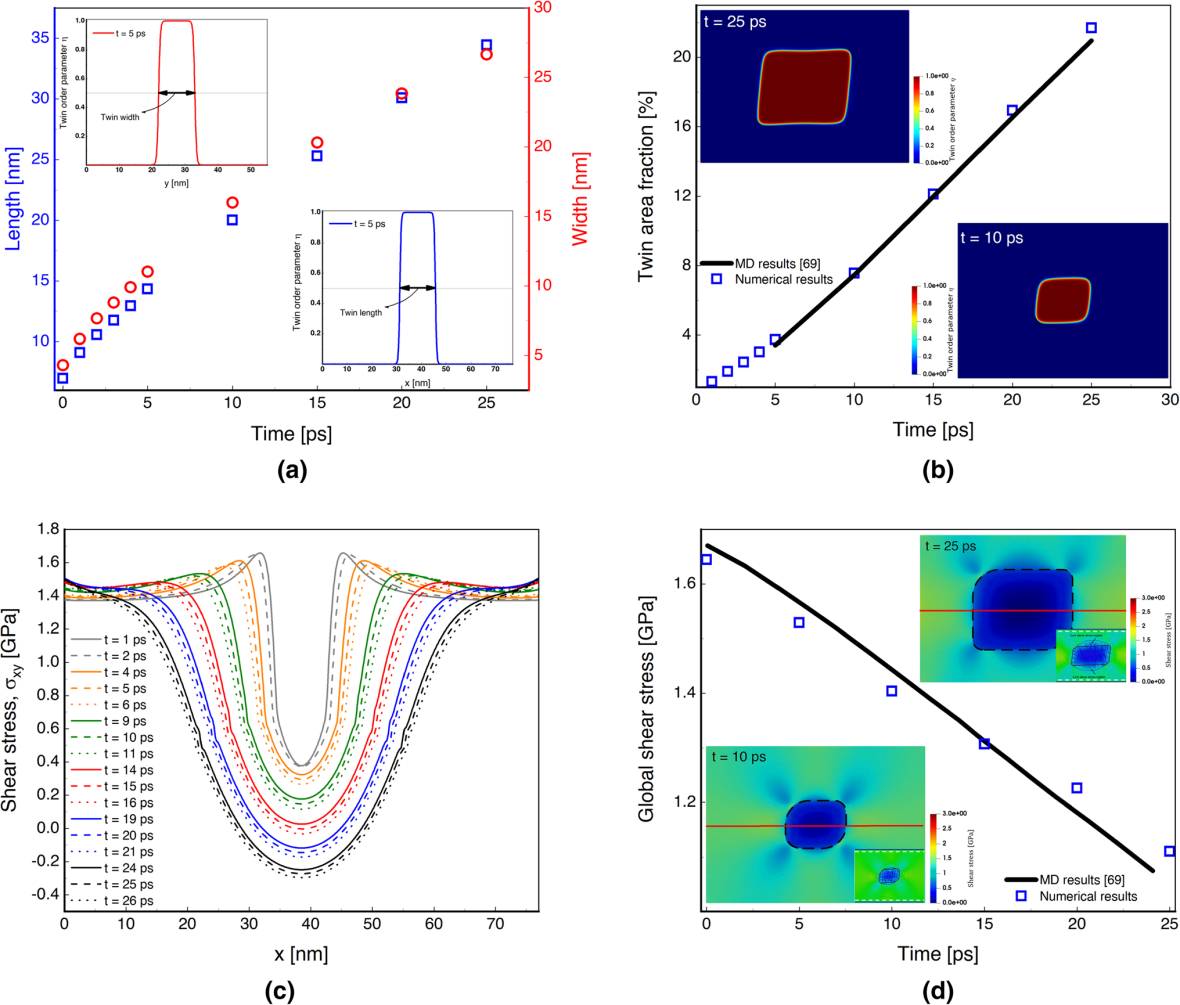
The time-evolved shear stress acquired from the phase-field model on deformation twinning of single-crystal pure magnesium. (a) Time evolution of the length (blue squares) and width (red circles) of a single rectangular twin embryo that grows at 7% shear strain. The insets show the twin interface profiles at $t = 5 \text { ps}$, parallel and orthogonal to the habit plane, by which the twin size is obtained; (b) Growth of the twin area fraction (i.e., the ratio of twinned area to the total area of the numerical geometry) predicted by the proposed phase-field approach (blue squares) and compared with molecular dynamics simulations (black line) [69]. The same numerical geometry setup as [69] was used. The insets show the distribution of the twin order parameter at $t = 10 \text { ps}$ and $t = 25 \text { ps}$ to illustrate areal growth; (c) Spatial variation of initial shear stress along the *x*-axis in single-twinned magnesium at various time instants; (d) Variation of the global shear stress as a function of time. The numerical results (blue squares) are compared with molecular dynamics data (black line) [69]. The insets show the spatial distribution of local shear stress at $t = 10 \text { ps}$ and $t = 25 \text { ps}$ along the red mid-line. The boundaries of the twin embryo are denoted by the black dashed line. In the bottom of each insets, the atomic shear stress from snapshots taken at similar times as [69] are given for comparison. (For interpretation of the references to color in this figure, the reader is referred to the web version of this article.)

**Fig. 4:**
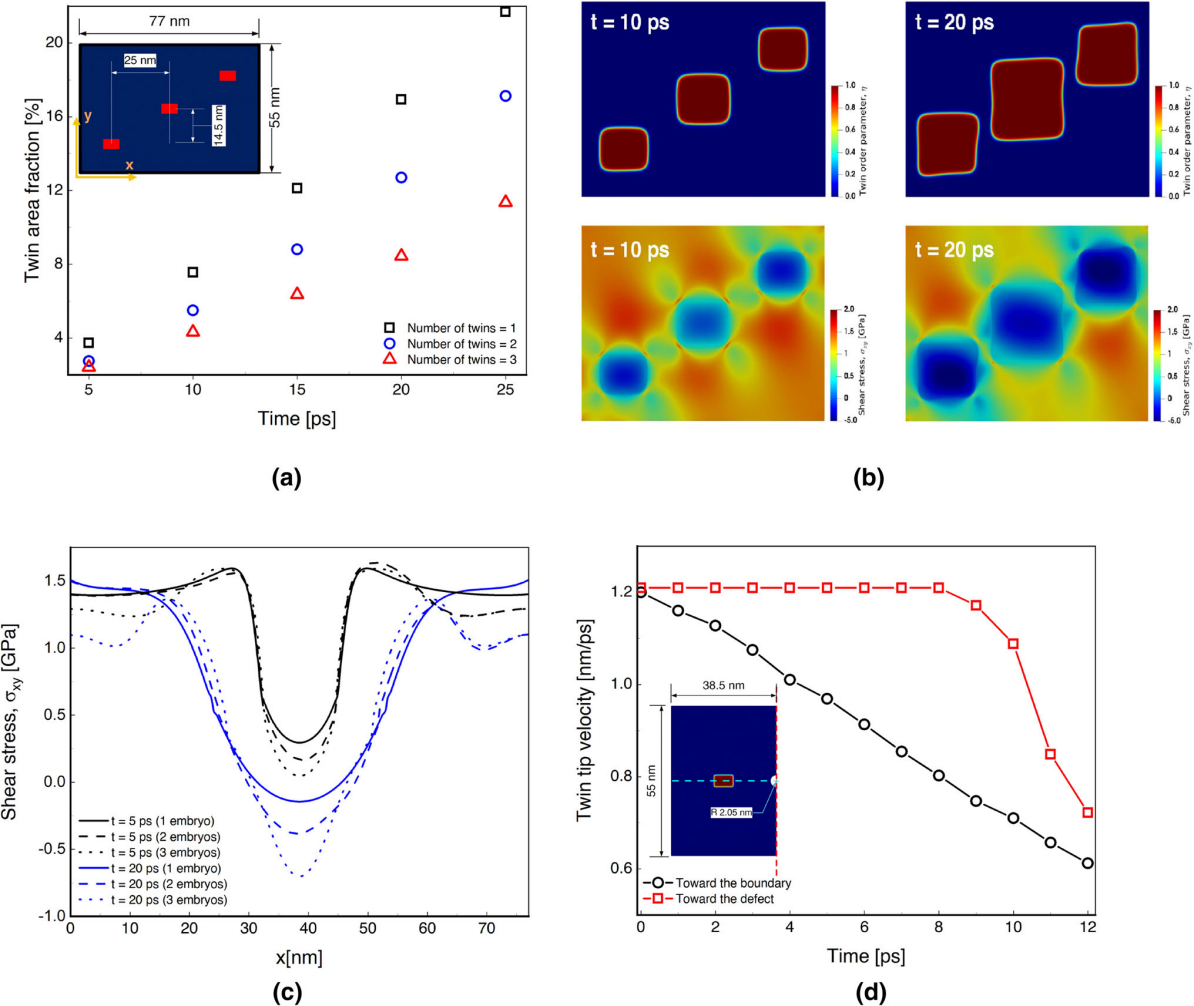
Exploration of twin-twin and twin-defect interactions to inform fundamental growth mechanisms in single crystal magnesium. (a) Evolution of twin area fraction for 1, 2, and 3 twin embryos. The inset shows the location of each twin for the three-embryo simulation. The area of the middle twin is measured using its length and width obtained from the interface profile at $$\eta = 0.5$$, as was done for Fig. 2; (b) Spatial distribution of the twin order parameter and shear stress in the parent and twin phases for the numerical setup shown in the inset of (a) at $$t=10 $$ and $$t=20 \text { ps}$$; (c) Evolution of the shear stress along a horizontal line through the middle of the single crystal microstructure for different numbers of embryos. The numerical setup is subjected to 7% shear strain as was done in the other examples; (d) Study of twin-defect interactions by considering the time-evolved twin tip interface towards the boundary and the void. The related simulation dimensions are given in the inset, which also shows that symmetric boundary conditions were used (the symmetry line is shown by the dash red line). (For interpretation of the references to color in this figure, the reader is referred to the web version of this article.)

We verify the proposed implementation of the time-resolved continuum-based model for magnesium by the static phase-field model [68] and molecular dynamics (MD) simulations [69] (Fig. 1). By choosing the same length-scale for the phase-field model and MD simulations, we assure the compatibility of MD results with our implementation, which is often left aside in the literature [51, 56, 60]. It is also worth stating that all MD simulations use extremely high deformation rates, making it difficult to understand whether a phenomenon results from the rate sensitivity of the material or is a numerical artifact [70, 71]. To remedy this, various strategies can be used to bridge the gap between the atomic scale and continuum frameworks, such as large-scale MD calculations [72], coarse-graining [73], and ultra-high strain-rate tests [74]. Twin propagation speed is explored (Fig. 2) and compared with MD results [69] and analytical solutions [75]. In this way, we demonstrate a simple yet effective approach on how to determine the mobility parameter. Moreover, insights in growth rates are of interest given the limited available data [27] and studying these behaviors is vital in high-rate applications of magnesium [76]. Our presented results are then validated in terms of twin area fraction and global shear stress (Fig. 3), and the role of twin-twin and twin-defect interactions is explored (Fig. 4). Through these approaches, the research offers broad potential in materials design, and motivates promising directions in experimental and computational materials science.

## Governing equations

We use standard continuum mechanics notation, where Latin indices refer to spatial coordinates. We use Einstein’s summation convention over repeated indices. All tensors are expressed in Cartesian coordinates. The superscripts $\text {E}$ and $\text {IE}$ stand for elastic (recoverable) and inelastic (irreversible) deformations, respectively. For the description of the twin, an order (phase-field) parameter, $\eta$, is introduced, where $\eta = 0$ denotes the parent crystal and $\eta = 1$ means the twin. This order parameter as well as displacement, $\varvec{u}$, are the primitive variables in space and time that we are searching for. The deformation gradient reads

1 $$\begin{aligned} F_{ij} = u_{i,j} + \delta _{ij}, \end{aligned}$$

where comma denotes a derivative in space. We use a material frame, where the derivative is taken in the reference configuration that is chosen to be the initial placement of the continuum body. Kronecker delta, $\varvec{\delta }$, is the identity. The deformation gradient, $\varvec{F}$, in a large-displacement formulation, is decomposed into elastic and inelastic parts,

2 $$\begin{aligned} F_{ij} = F^\text {E}_{ik} F^\text {IE}_{kj}, \end{aligned}$$

where for (inelastic) twinning [77], we use

3 $$\begin{aligned} F_{ij}^\text {IE}=\delta _{ij}+\phi (\eta )\gamma _{0} s_i m_j . \end{aligned}$$

The interpolation function, $\phi (\eta )=\eta ^{2}(3-2\eta )$, causes a steep change between the twin and parent crystal [78] as necessary in phase-field approaches. $\gamma _{0}$ is the magnitude of maximum twinning shear, and $\varvec{s}$ and $\varvec{m}$ are the unit vectors along the twinning direction and normal to the twinning plane, respectively. By following [79], we decompose the Helmholtz free energy per mass into mechanical and interfacial parts,

4 $$\begin{aligned} \psi (\varvec{F}, \eta , \nabla \eta ) = \psi ^\text {M}(\varvec{F}, \eta ) + \psi ^\nabla (\eta , \nabla \eta ) \ , \end{aligned}$$

where the kinetics of interface is controlled by twin order parameter and its first-gradient by the latter. As usual, for the mechanical deformation energy density (per volume), we may use the St. Venant model:

5 $$\begin{aligned} \rho _0 \psi ^\text {M} = \frac{1}{2} E_{ij} C_{ijkl} E_{kl} \ , \end{aligned}$$

or the neo-Hookean model:

6 $$\begin{aligned} \rho _0 \psi ^\text {M} = \frac{\mu }{2} \left( I_C - 3 \right) - \mu \ln {J} + \frac{\lambda }{2} \left( \ln {J}\right) ^{2} \ . \end{aligned}$$

For nonlinear isotropic elasticity, the neo-Hookean model defined in Eq. (6) is used. We use right Cauchy–Green deformation tensor, $C^\text {E}_{ij} = F^\text {E}_{ki} F^\text {E}_{kj}$, and its invariants, $I_C = C^\text {E}_{ii}$, $J=\det (\varvec{C}^\text {E})$. The Green–Lagrange strain measure, $\varvec{E} = \frac{1}{2}(\varvec{C}^\text {E}- \varvec{\delta })$, accommodates geometric nonlinearity necessary for some applications herein. Lamé parameters, $\lambda$, $\mu$, or the stiffness tensor of rank four, $C_{ijkl}$, are given as material coefficients. The elastic constants are the Voigt-averaged shear and bulk modulus [80], which are listed in Table 1. For anisotropic elasticity, the elastic coefficients are interpolated between the untwinned $C^\text {P}_{ijkl}$ and twinned $C^\text {T}_{ijkl}$ domains using the interpolation function,

7 $$\begin{aligned} C_{ijkl} = C^\text {P}_{ijkl} + ( C^\text {T}_{ijkl} - C^\text {P}_{ijkl} ) \phi (\eta ) \ . \end{aligned}$$

The same interpolation function is used as in the definition of the inelastic part of the deformation gradient. For the twin phase, $\eta =1$, we have the stiffness tensor as a rotation of crystal lattice from the parent phase, $\eta =0$, as follows:

8 $$\begin{aligned} C_{ijkl}^\text {T} = {\mathcal {Q}}_{im}{\mathcal {Q}}_{jn}{\mathcal {Q}}_{ko}{\mathcal {Q}}_{lp} C_{mnop}^\text {P} , \end{aligned}$$

where $\varvec{{\mathcal {Q}}}$ is the reorientation matrix associated with twinning. For a centrosymmetric structure [81], it becomes

9 $$\begin{aligned} {\mathcal {Q}}_{ij} = {\left\{ \begin{array}{ll} 2 m_{i} m_{j} - \delta _{ij} &{} \text {type I twins},\\ 2 s_{i} s_{j} - \delta _{ij} &{} \text {type II twins}. \end{array}\right. } \end{aligned}$$

In the case of a steady-state deformation and neglecting inertial terms, the governing equations for displacement read

10 $$\begin{aligned} \begin{aligned} P_{ji,j} =&0 \ ,\\ P_{ji} =&\frac{\partial \rho _0 \psi }{\partial F_{ij}} = \frac{\partial \rho _0 \psi ^\text {M}}{\partial F_{ij}} = \frac{\partial \rho _0 \psi ^\text {M}}{\partial E_{kl}} \frac{\partial E_{kl}}{\partial F_{ij}} \\ =&\frac{\partial \rho _0 \psi ^\text {M}}{\partial E_{kl}} F^\text {E}_{il} (\varvec{F}^\text {IE})^{-1}_{jk} \ . \end{aligned} \end{aligned}$$

The Ginzburg–Landau equation is acquired by a thermodynamically-consistent derivation, as follows:

11 $$\begin{aligned} \begin{aligned} \dot{\eta } =&-{\mathcal {L}} \Bigg ( \frac{\partial \rho _0 \psi ^\text {M}}{\partial \eta } + \frac{\partial \rho _0 \psi ^\nabla }{\partial \eta } - \left( \frac{\partial \rho _0 \psi ^\nabla }{\partial \eta _{,i}}\right) _{,i} \Bigg ) \ , \end{aligned} \end{aligned}$$

where the mobility parameter, ${\mathcal {L}}$, is generally not known and challenging to obtain experimentally. The outcome of this work is the methodology on how to set its numerical value.

The first term is formulated by using the product rule

12 $$\begin{aligned} \frac{\partial \rho _0 \psi ^\text {M}}{\partial \eta }&= \frac{\partial \rho _0 \psi ^\text {M}}{\partial F_{ij}} \frac{\partial F_{ij}}{\partial \eta } = P_{ji} \frac{\partial F^\text {E}_{ik} F^\text {IE}_{kj} }{\partial \eta } \nonumber \\&= P_{ji} F^\text {E}_{ik} \phi '(\eta ) \gamma _0 s_k m_j \ , \end{aligned}$$

where $\phi '(\eta )=6\eta (1-\eta )$. For the interfacial energy, $\psi ^{\nabla }$, we use a standard double-well potential as in [82, 83] such that the energy density reads

13 $$\begin{aligned} \rho _0 \psi ^{\nabla }(\eta )=A\eta ^{2}\left( 1-\eta \right) ^{2} + \kappa _{ij} \eta _{,i}\eta _{,j} , \end{aligned}$$

where $A=12\frac{\Gamma }{l}$ characterizes the energy barrier between two stable phases (minima), related to the twin boundary surface energy, $\Gamma$, and the twin boundary thickness, *l*; $\kappa _{ij}=\kappa _{0}\delta _{ij}$ with $\kappa _{0}$ being the gradient energy parameter, given as [68], $\kappa _{0} = \frac{3}{4} \Gamma l$. By inserting the energy definitions into the Ginzburg–Landau, we obtain the governing equation for twin order parameter,

14 $$\begin{aligned} \dot{\eta }&= -{\mathcal {L}} \Big ( P_{ji} F^\text {E}_{ik} \phi '(\eta ) \gamma _0 s_k m_j + 2 A \eta \big ( 1-3\eta + 2\eta ^{2} \big )\nonumber \\&\quad - 2\kappa _{0}\eta _{,ii} \Big ) \end{aligned}$$

By solving Eqs. (10), (14), we obtain $\varvec{u}$ and $\eta$ fields.

## Computational implementation

The presented numerical simulations employ a monolithic strategy in order to solve Eqs. (10), (14). Because of their inherent coupling, a monolithic solution method is preferable for capturing all effects accurately, especially in extreme loading conditions. Mostly, a staggered scheme is implemented partly to increase efficiency yet also effected by numerical difficulties in implementing as a monolithic strategy. Herein, we use the interface energy as described above, which helps to circumvent any numerical convergence errors in the implementation. In a monolithic scheme, for each time step, displacements and order parameter are solved at once. Therefore, for the space discretization, we use an adequate mixed space formulation in the implementation. Specifically, we use $\varvec{u}$ and $\eta$ as approximated functions spanned over a triangulation with a compact support. This well-known finite element method (FEM) ensures a monotonic convergence for the implementation. We skip a notational distinction between the analytical functions and their approximations since they never show up together.

The computational domain, $\Omega$, is the continuum body’s image in the physical space. The domain, $\Omega$, and its closure as a Lipschitz boundary, $\partial \Omega$, form a continuous domain without singularities. Therefore, all form functions are continuous as well. Triangulated domain in finite number of nodes is representing the approximated unknown functions, $\varvec{u}$ and $\eta$, with the interpolation between the nodes by the form functions, as follows:

15 $$\begin{aligned} {\mathscr {V}} =\Bigg \{ \big \{ \varvec{u}, \eta \big \} \in [ {\mathscr {H}}^{n}(\Omega ) ]^\text {DOF} : \big \{ \varvec{u}, \eta \big \} = \text {given} \ \forall \varvec{x} \in \partial \Omega _\text {D} \Bigg \}.\nonumber \\ \end{aligned}$$

The Hilbertian-Sobolev space, ${\mathscr {H}}^n$, is of polynomial order, *n*, hence, we use standard Lagrange elements in the FEM [84]. On each node, we have $2+1=3$ degrees of freedom (DOFs) in two-dimensional and $3+1=4$ (DOFs) in three-dimensional spaces. As known as the Galerkin approach, the test functions, $\updelta \varvec{u}$ and $\updelta \eta$, are approximated by the same mixed space. They vanish on Dirichlet boundaries, $\partial \Omega _\text {D}$, where the solution, $\varvec{u}$ or $\eta$, is given. For other boundaries, we use Neumann boundary condition, for displacement, $\varvec{u}$, it denotes the given traction vector, $\hat{\varvec{t}}$, and for twin order parameter, $\eta$, we implement zero Neumann boundaries meaning that the twin phase fails to leave the boundary across boundaries. The latter is justified easily since the twin or parent phase is neither convective nor conductive. The twin growth is inhibited by the displacement boundary conditions. The twin order parameter gradient also vanishes at the boundaries due to the Neumann boundary condition.

For time discretization, we use constant time steps in order to be able to determine an adequate time step by a convergence analysis. Given the data at a time instant, $t^{n}$, we solve $\varvec{u}$ and $\eta$ by a standard variational formulation leading to a weak form. The time derivative of order parameter is discretized using a so-called $\theta$-scheme, for an arbitrary field, $y^n=y(t^n)$ and $y^{n-1}=y(t^{n-1})$, we use

16 $$\begin{aligned} y^{n-\theta } = (1-\theta ) y^{n-1} + \theta y^{n} \ . \end{aligned}$$

This scheme requires the computed solution from the last time step, $y^{n-1}$, by evaluating the functions within the time step, leading to a higher accuracy in the discretization [85]. For $\theta =0$, this method is the first-order accurate explicit Euler method. For $\theta =1$, it becomes the first-order accurate implicit Euler method. For $\theta =0.5$, we obtain the second-order accurate Crank–Nicolson method. We use the time discretization in Eq. (14) for one finite element $\Omega ^\text {e}$, as follows:

17 $$\begin{aligned}&\int _{\Omega ^\text {e}} \Bigg ( \frac{\eta ^n - \eta ^{n-1}}{\Delta t} + {\mathcal {L}} \bigg ( P_{ji} F^\text {E}_{ik} \phi '(\eta ^{n-\theta }) \gamma _0 s_k m_j \nonumber \\&\quad + 2 A \eta ^{n-\theta } \Big ( 1-3\eta ^{n-\theta }+ 2(\eta ^{n-\theta })^{2} \Big )\nonumber \\&\quad - 2\kappa _{0}\eta ^{n-\theta }_{,ii} \bigg ) \Bigg ) \updelta \eta \, \text { d} V = 0 \ . \end{aligned}$$

The test function, $\updelta \eta$, may have a lower continuity than the trial function, $\eta$, but we stress that we aim for the Galerkin procedure such that they are chosen from the same mathematical space. In order to weaken the continuity condition on $\eta$, we integrate by parts terms of second gradient,

18 $$\begin{aligned}&\int _{\Omega ^\text {e}} \Bigg ( \frac{\eta ^n - \eta ^{n-1}}{\Delta t} \updelta \eta + {\mathcal {L}} P_{ji} F^\text {E}_{ik} \phi '(\eta ^{n-\theta }) \gamma _0 s_k m_j \updelta \eta \nonumber \\&\quad + {\mathcal {L}} 2 A \eta ^{n-\theta } \Big ( 1-3\eta ^{n-\theta } + 2(\eta ^{n-\theta })^{2} \Big ) \updelta \eta \nonumber \\&\quad + 2 {\mathcal {L}} \kappa _{0}\eta ^{n-\theta }_{,i} \updelta \eta _{,i} \Bigg ) \, \text { d} V- \int _{\partial \Omega ^\text {e}} 2 {\mathcal {L}} \kappa _{0}\eta ^{n-\theta }_{,i} n_i \, \text { d} A = 0 .\nonumber \\ \end{aligned}$$

By summing over each element, on each boundary of elements we sum twice with neighboring elements’ surface normal directed oppositely. Therefore, we obtain a jump condition, which we enforce to vanish by setting it zero. In other words, the weak formulation searches for a continuous $\eta _{,i} n_i$ across element boundaries resulting in a smooth phase change within the finite element. In this way, a mesh dependency is prevented as long as the element size is adequately small such that the numerical result is converged. On the boundaries of the whole domain, we assume zero Neumann boundaries meaning that $\eta$ is not leaving the domain across the outer boundary. Hence, we obtain for $\Omega = \bigcup \Omega ^\text {e}$, the following weak form:

19 $$\begin{aligned} \text {Form}_\eta= & {} \int _{\Omega } \Bigg ( \frac{\eta ^n - \eta ^{n-1}}{\Delta t} \updelta \eta + {\mathcal {L}} P_{ji} F^\text {E}_{ik} \phi '(\eta ^{n-\theta }) \gamma _0 s_k m_j \updelta \eta \nonumber \\&\quad + {\mathcal {L}} 2 A \eta ^{n-\theta } \Big ( 1-3\eta ^{n-\theta } + 2(\eta ^{n-\theta })^{2} \Big ) \updelta \eta \nonumber \\&\quad + 2 {\mathcal {L}} \kappa _{0}\eta ^{n-\theta }_{,i} \updelta \eta _{,i} \Bigg ) \, \text { d} V . \end{aligned}$$

Analogously, from Eq. (10), we obtain the weak form for displacement, where the traction $t_i = n_j P_{ji}$ is enforced to be continuous across the element. This so-called Newton’s second lemma is a basic assumption for regular domains (no singularities). On outer boundaries, for Dirichlet boundaries, where displacement is given, the test function vanishes and we allow for Neumann boundaries that traction vector, $\hat{\varvec{t}}$ in Pa, is given. The weak form for displacements, $\varvec{u}$, reads

20 $$\begin{aligned} \text {Form}_{\varvec{u}} = -\int _\Omega P_{ji} \updelta u_{i,j} \, \text { d} V + \int _{\partial \Omega _\text {N}} {\hat{t}}_i \updelta u_i \, \text { d} A \ . \end{aligned}$$

The objective is to solve both fields as unknowns, $\varvec{p} = \{ \varvec{u}, \eta \}$, at once by satisfying

21 $$\begin{aligned} \text {Form}_\eta + \text {Form}_{\varvec{u}} = 0 \ . \end{aligned}$$

The weak form is nonlinear. We use a standard Newton–Raphson linearization method, where the weak form is used to get a Jacobian by a derivative with respect to unknowns, $\varvec{p}$. High-level tools are exploited to generate computer code automatically by performing a symbolic differentiation for this linearization. In this manner, use of different stored energy models is indeed possible without major changes in the implementation. We use software packages from the FEniCS Project [86, 87]. The time stepping parameters are chosen such that the momentum balance scheme is second-order accurate and stable. Quadratic and linear Lagrange functions are used for the finite element approximation of the displacement and the twin order parameter, respectively. The conjugate gradient method with a Jacobi preconditioner from PETSc packages [88] has been employed for solving the nonlinear equations. The simulation has been performed by a computing node using Intel Xeon E7-4850, in total 64 cores each with the 40 MB cache, equipped with 256 GB Memory in total, running Linux Kernel 5 Ubuntu 20.04.

## Results and discussion

The material parameters are compiled from different sources and given in Table 1. For anisotropic cases, we use the stiffness tensor with the given components and isotropic cases the Lamé constants, $\lambda$, $\mu$. The computational domain is a 2-D rectangular shape at nanometer (nm) length-scale. Accordingly, units are chosen to be nanonewton (nN) and picosecond (ps). A mesh of 423 500 triangular elements is adopted. Initial conditions are prescribed as zero displacement and a given twin/parent phase field, which is described in each example. It is noted that 10 elements are considered at the interface to resolve the sharp variation along the interface width.

**Table 1:** Material properties and model constants for single crystal magnesium compiled from [16, 25, 69, 82, 89]

Parameters	Notation	Value
Second order elastic constants	$C_{11}=$	$63.5\text { GPa}$
	$C_{12}=$	$25.9\text { GPa}$
	$C_{13}=$	$21.7\text { GPa}$
	$C_{33}=$	$66.5\text { GPa}$
	$C_{44}=$	$18.4\text { GPa}$
Bulk modulus	$K=$	$36.9\text { GPa}$
Shear modulus	$\mu =$	$19.4\text { GPa}$
Poisson’s ratio	$\nu =$	0.276
Twin boundary surface energy	$\Gamma =$	$0.117\text { J/m}^{2}$
Twinning shear for $\langle {1 0 \bar{1} 1}\rangle \{\bar{1}$ 0 1 2}	$\gamma _0=$	0.1295
Regularization length	$l=$	$1.0\text { nm}$
Transformation barrier	$A=$	$1.404\text { GPa}$
Gradient energy parameter	$\kappa _{0}=$	$0.0878\text { nJ/m}$
Ginzburg–Landau kinetic factor	${\mathcal {L}}=$	$4200(\text {Pa}\text { s})^{-1}$

### Validation of the phase-field model and twin order parameter for single crystal magnesium

We validate our time-resolved phase-field model for single crystal magnesium using previous static phase-field results [68] and molecular dynamics simulations [69] (Fig. 1). The presence of pronounced mechanical anisotropy, local stress concentrations, and high pressure in nanoscale defect-free magnesium implies employing ansiotropic mechanical properties, anisotropic surface energy, and a large displacement formulation in our simulations. The nucleation and evolution of deformation twinning in a magnesium single crystal is simulated using the same initial twin geometry as in [68]. A circular twin embryo of initial radius $r = 3 \text { nm}$ (corresponding to the analytical sharp interface solution [90]) is embedded into a rectangular domain of dimensions $40 \text { nm} \times 40 \text { nm}$ in plane strain conditions. The $\langle {1 0 \overline{1} 1}\rangle$ plane and $\{\overline{1} 0 1 2\}$ directions are considered as the primary twinning system [13]. Consequently, there is no need to assume the dependency of the mobility parameter to the angle between the direction normal to the interface and a specified direction in crystal as well as temperature, due to the fact that the kinetic coefficients differ by only about 1% in different planes and directions [91]. The validation simulations in Fig. 1 are performed to investigate the twin parameter distribution subject to simple shear with Dirichlet boundary conditions on the order parameter for different cases, including an isotropic (Fig. 1(a, c, d, g, h)) and an anisotropic surface energy (Fig. 1(b, e, f, i, j)) at three different time instants. Within the simulation time of $500 \text { ps}$, the twin embryo grows until it is repelled by the rigid outer boundaries. For the anisotropic case, the equilibrium shape of the twin embryo is wider in the horizontal direction (parallel to the habit plane) and flatter normal to the habit plane when compared with the isotropic case, which is in good qualitative agreement with the reference phase-field results [68] shown in Fig. 1(m). In addition, the twin interface thickness has a lower value normal to the habit plane for the anisotropic surface energy when compared with the ideal isotropic one. This may be related to the contribution of the core and elastic energies to the total surface energy of the interface [92]. For large deformation simulations (Fig. 1(b, d, f, h, j)), an orientation of the twin evolution is realized due to the difference in the driving force for twinning, which is a factor of $\left( \varvec{F}^{\eta }\right) ^{-1}$.

Overall, the twin shape predicted by the current time-dependent phase-field approach shows features in good agreement with the molecular dynamics simulation [69] (Fig. 1(k)) and steady-state continuum-based model [68] (Fig. 1(l, m)). Finally, it is worth mentioning that the twin tends to shrink and eventually disappear when the magnitude of the shear loading was lower than $\gamma _{0} = 0.07$ or the size of the initial nucleus were lower than $3 \text { nm}$. This detwinning mechanism has been observed previously in copper [93] and gold nanowires [94], but this is not the focus of the present contribution.

### The determination of the kinetic coefficient, ${\mathcal {L}}$, for magnesium using twin tip and twin boundary velocities

The kinetic coefficient or mobility parameter, ${\mathcal {L}}$, plays an important role in describing the twin propagation and its dependence on other parameters (e.g., shear stress) during the early stages of twin morphology [95, 96]. Experimental studies lack a quantification of the twin boundary mobility in magnesium since the evolution is too fast for obtaining an adequate measurement. In order to address this, we propose to determine ${\mathcal {L}}$ for single crystal magnesium by using interface velocity profiles in both twin tip and twin boundary directions by comparing the present time-resolved phase-field results with molecular dynamics simulations [69] (Fig. 2). Here, we assume that the molecular dynamics solution represents a reliable experiment and we try to find the kinetic coefficient such that we obtain matching results. Considering a single twinning plane and direction as the primary deformation mechanism, an isotropic kinetic coefficient is obtained for predicting the microstructure evolution in two-dimensional single crystal magnesium at room temperature. This assumption is consistent with the other atomistically informed phase-field model [52, 55]. Although, taking into account an anisotropic kinetic coefficient which depends on free energy functional parameters (e.g., temperature or interface orientation) is required to accurately describe the other phase transformation (e.g., liquid-liquid, liquid-vapor, and solid-melt phase transformations) interface kinetics [97]. A rectangular twin embryo with an initial length of $7 \text { nm}$ and width of $4.3 \text { nm}$ inserted at the center of a $77 \text { nm} \times 55 \text { nm}$ rectangular domain as in Fig. 2(a). The domain is under simple-shear, the $\left( \overline{1} 0 1 2 \right)$ twinning planes (i.e., the horizontal planes) are referred to as twin boundaries (TB), and the $\left( 1 0 \overline{1} 2\right)$ twinning planes (i.e., the vertical planes) are referred to as twin tips (TT). Applying the shear deformation in the $[1 0 \overline{1} 1]$ direction results in the twin interface profiles illustrated in Figs. 2(b) and 2(c) for the twin boundary and twin tip for times noted in the sub-figures, respectively. The twin boundary and twin tip velocities are calculated by tracking the horizontal, $\Delta x$, and vertical, $\Delta y$, interface displacement of the planes of the twin at $\eta = 0.5$ over time—along the green line in Figs. 2(b) and 2(c). The results indicate that the twin boundary (black color) and twin tip (blue color) velocities are decreasing and constant, respectively, with values of velocity summarized in Fig. 2(d). The constant velocity trend of twin tip mobility may be ascribed to the large back-stress arising at the twin tip [95]. Mapped in red onto Fig. 2(c) is the explicit analytical solution for the stationary Ginzburg–Landau equation given by [75]

22 $$\begin{aligned} \eta _{\text {analytical}} = \left( 1 + \exp \Big (\frac{-x}{w} \Big )\right) ^{-1}; \ \ \ w=\sqrt{\frac{\kappa _{0}}{2A}}. \end{aligned}$$

The comparison of numerical results with this analytical solution enables the twin interface width (i.e., difference between twin interface position at $\eta = 0.01$ and $\eta = 0.99$) to be calculated. The determination of the twin interface width is important because its size can guide the selection of the element size and spatial mesh refinement in finite element simulations of twinning [83].

Altogether, Fig. 2 provides a good validation for the present time-dependent phase-field approach, and, more importantly, enables the first ever determination of the kinetic energy coefficient, ${\mathcal {L}} = 4200~(\text {Pa } \cdot \text { s})^{-1}$, for single crystal magnesium.

### The time-evolved shear stress in the combined matrix-twin embryo

For a better comprehension of the underlying mechanism, we study the evolution of the twin area fraction and the shear stress, $\sigma _{12}$, in the parent and twin phase (Fig. 3). Local stress distribution within a small region in the microstructure is understood as the driving force for the propagation and growth of a twin. These insights may inform about the sequence of events leading to the formation of the visible twins at an early stage in magnesium.

In Fig. 3, the same boundary conditions and a constant 7% shear strain are used in the same rectangular twin embryo system depicted in Fig. 2(a). Initially, the length and width of a single rectangular twin embryo at different times are calculated in Fig. 3(a); this will be used to obtain the twin area fraction in Fig. 3(b). In the figure, values are calculated for $\eta = 0.5$ on the interface profile as shown in the insets at $t = 5 \text { ps}$. Results indicate that the twin growth is larger in the twin tip direction rather than in the twin boundary direction, and this difference decreases at later time instants as the twin approaches the outer boundaries.

Next, the change of the twin area fraction, defined as the ratio of the twinned to the whole simulated area, is shown in Fig. 3(b) under shear loading, and this is compared with molecular dynamics simulations [69]. The insets in Fig. 3(b) show the morphology of the twin at two different times for visualizing how the twins grow. Knowing the twin area fraction evolution is important towards enhancing our understanding of the crystal grain reorientation associated with deformation twinning, where limited data exists because of the special experimental tools needed to access the length and time scales needed to capture such measurements [27]. As seen in Fig. 3(b), the present phase-field model reasonably predicts the evolution of the twin area fraction. Next, the shear stress profile acting parallel to the *x*-direction is plotted for various times in Fig. 3(c), which is used to demonstrate the redistribution of internal stresses resulting from twinning [98]. The plateau and decreasing regions indicate the shear stress variation in the parent and twin phases, respectively. By progressing in time, the shear stress decreases as the *x*-position approaches the center of the simulation geometry, until it reaches its minimum. The magnitude of the shear stress within the twin decreases as a function of time and, eventually, becomes negative for the last time instants of the simulation. This phenomenon is consistent with experimental results [17]. At the same time, the profile evolves spatially and temporally.

Finally, the global shear stress field is shown in Fig. 3(d), where the field is taken as the average across the red line spanning both the twin and the matrix depicted in the inset. The measurements are important because they can provide insights into the complex load sharing mechanisms that are generated by the parent and the twin phase [99]. The results are also compared with molecular dynamics simulations [69], both qualitatively (the insets at $t=10 \text { ps}$ and $t=25 \text { ps}$) and quantitatively. The phase-field results match the molecular dynamics simulations well. The results show that the global shear stress is decreasing as the twin size evolves. Altogether, results from Fig. 3 are important for determining the activation force required for twin embryo growth that may serve as an input into higher scale models [100].

### Studying twin interactions toward microstructure tailoring and materials design

Finally, simulations have been performed to study the effect of twin-twin and twin-defect interactions (Fig. 4). Understanding these interactions is an important step toward developing better predictive models for designing materials with tailored properties [101–104] and microstructures [105–108]. Damage in materials is studied by phase-field models [109–113], and we use phase-field approach herein for twin interactions. These interactions [114] may result in the formation of twin-twin junctions that may cause strain hardening [115] and crack initiation [116, 117], leading to a strong influence on the overall material performance. First, the change of area fraction of the middle twin as a function of time for a different number of embryos is illustrated in Fig. 4(a). Only the middle embryo is considered in the analysis in order to better isolate the interactions and reduce boundary effects. The location of the twins for the three embryo cases is illustrated in the inset. In Fig. 4(a), it is shown that increasing the number of twins leads to a decrease in the twin area fraction of the middle embryo as a result of its interaction with the other twins. The difference of the twin area fraction for multi-embryo cases becomes larger at later time instants. This finding is important as it highlights the effects of twin interactions on twin evolution, where experimental measurements are currently very limited [118]. Next, the spatial variation of the order parameter and the corresponding shear stress at $$t = 10 $$ and $$t = 20 \text { ps}$$ are depicted in Fig. 4(b). This result reveals insights into the expansion of the twin domain through the accumulation of large plastic shear strain at the nano-scale [119].

The homogeneous growth in the twin area is exemplified in the top left inset in Fig. 4(b), where the twins have not changed in shape until $t = 10 \text { ps}$. The corresponding shear stress distribution at $t = 10 \text { ps}$ is shown in the bottom left inset, where the shear stress inside the twins is negative while it is positive in the matrix. The heterogeneous stress distribution around the twins is due to a sudden change in the stresses within the twin interfaces, associated with the need to accommodate deformation in this region [40]. From the spatial shear stress distribution, it is observed that the local shear stress reaches a minimum in the center of each twin. Outside the twins, the shear stress is lower at the bottom left and top right twins because of the constraining effect of the adjacent twins to the middle one. In the right insets, the deviatoric deformation in twin morphology at $t = 20 \text { ps}$ is identified due to the interaction of the twins with each other and the disturbing of the stress field by them. The stress distribution in the vicinity of the twin-matrix interfaces at $t=20 \text { ps}$ is heterogeneous as a result of high stress concentrations in the matrix near the twin boundaries. It is also shown that the middle twin experiences a maximum shear stress resulting from the compressive forces generated by the other twins. The local stress concentration is one main interaction of crack and twins where some nucleation site appears in the interfaces inside and around the interface [120].

Next, the change of shear stress along a horizontal line through a middle section of the simulation area as a function of a 1, 2, or 3 embryo system is shown in Fig. 4(c). It is observed that increasing the number of twins leads to decreasing the shear stress values in the matrix phase, while the difference in shear stress values for the later time instants are larger as a result of twin-twin interactions. In the twinned regions at later times, the junctions of different embryos result in a negative shear stress with steeper slopes as compared with earlier times. In addition, it can be observed that the stress concentration in the matrix, predominantly in the vicinity of the twin boundaries, increases only marginally with increasing twin thickness (black lines in Fig. 4(c)). Finally, the interaction of a twin and a defect is investigated in Fig. 4(d) by comparing the change in the twin tip velocity towards the boundary and the void along the blue dashed horizontal line. The numerical setup is also given in the inset, where symmetric boundary conditions are used. The radius of $2 \text { nm}$ is chosen for the void. For all times, the results indicate that the tip velocity is linearly decreasing in time in a direction approaching the left boundary. For the void, the velocity at the tip is constant until some point after which a sudden decrease in the velocity occurs, resulting from the twin-defect interaction. In addition, the twin tip velocity is larger toward the void because of the higher stress concentration influenced by the void.

## Conclusions

In this paper, the evolution of twinning in magnesium has been studied using a validated and calibrated phase-field model to gain better insights into the time-evolved twin morphology, the spatial distribution of the internal shear stress, and the twin interactions. An accurate monolithic iterative procedure has been implemented for solving the coupled balance and Ginzburg–Landau equations, and the governing equations have been solved in the open-source high-level computing platform, FEniCS. For engineering examples with FEniCS, we refer to [121].

The results presented in this work confirmed the impact of the current model by capturing the behavior of the leading deformation mechanism in single crystal magnesium, twinning. By means of the proposed implementation, the state variables (i.e., the displacement and the twin order parameter) have been computed monolithically for various scenarios in discrete time steps, including small and large deformations with both isotropic and anisotropic surface energies and elasticity. The data have been compared with a continuum mechanics model [68] and molecular dynamics simulations [69]. The findings are qualitatively consistent with both literature approaches.

A notable result emerging from the proposed model is the prediction of the critical strain and initial twin embryo size required for growth and propagation under the chosen numerical settings. This computational implementation is particularly useful because identifying such features experimentally is challenging given the length and time scales needed to reproduce these events [122]. Next, the interface velocities for the twin tips and twin boundaries have been explored in order to determine the kinetic coefficient using the phase-field model and compared with recent molecular dynamics simulation [69]. Studying velocity growths is important because they affect hardening, texture evolution, and ductility in the material [123]. To the authors’ best knowledge, the present work pioneers the analysis of the interface mobility, showing different trends of twin evolution in the direction parallel and orthogonal to the twin habit plane.

The interface velocity is considered to be an important factor to determine the thermodynamic driving force for interface propagation, because knowing the interface velocity for any value of the driving force potentially leads to the determination of the kinetic coefficient for any range of materials [124]. The interface profile has been compared with the analytical solution of the stationary Ginzburg–Landau equation, and the obtained numerical interface width of $1.58 \text { nm}$ is close to the analytical value of $1.62 \text { nm}$ [75]. This information guides mesh selection and refinement when modeling twinning in this system [125, 126]]. In addition, the current phase-field modeling approach overcomes the challenges existing in molecular dynamic simulations for calculating the twin size, such as identifying the orientation of each atom in the twinned region [36], and is able to capture new behavior of twin growth for $t \le 5 \text { ps}$, comparing well with previous molecular dynamics data [69]. The strong point of the current approach is to track multiple interfaces in order to measure twins’ size with no additional efforts for samples larger or smaller than in atomistic simulations.

A further considerable implication of the proposed model is the possibility of investigating the local and global shear stress field inside the parent and twinned phases. Analysis of twin shear stress fields induced in these cases provides further evidence for the effect of twins’ thickness and their mutual position on further twin growth and/or further twin nucleation [127–129]. Moreover, the importance of an appropriate strategy for partitioning the stress fields between the twinned and untwinned domains have been demonstrated in this paper. A final upshot of the current phase-field model has been to explore new understandings in twin-twin and twin-defect interactions. For the case where multiple twins grow in one grain, a common occurrence observed in experiments [130], it is highlighted that the stress concentration around the void may significantly increase the twin interface velocity, affecting subsequent expansion of the twins. Taken together, our study provides a framework for a new way to understand local deformation mechanisms in materials by analyzing the evolution and interaction of twins.

## Data Availability

The authors declare that the main data supporting the findings of this study are available within this article. Extra data are available from the corresponding authors upon reasonable request.

## Code availability

The Python code, generated during the current study, is part of the FEniCS project available at http://www.fenicsproject.org/download, and an example for the computational implementation is available in [131] to be used under the GNU Public license [132].
